# Supplementary material for: A quantitative modelling approach to zebrafish pigment pattern formation
Source: eLife. 2020 Jul 27;9:e52998. doi: 10.7554/eLife.52998 (PMC7384860; doi:10.7554/eLife.52998)
Supplement: Supplementary file 1. [file elife-52998-supp1.pdf]

Supplementary File 1: This table summarises all of the notation used in Appendix 1.

**Symbol**	**Description**	**Definition**
M	Melanocyte.	
I^l	Loose form iridophore.	
I^d	Dense form iridophore.	
X^b	Xanthoblast.	
X	Xanthophore.	
$\boldsymbol{X}$	A representation of the xanthophore layer.	A $\Pi^L_{\boldsymbol{X}}(t) \times \Pi^H_{\boldsymbol{X}}(t)$ matrix of X's, X^b's and zeros.
$\boldsymbol{M}$	A representation of the melanocyte layer.	A $\Pi^L_{\boldsymbol{M}}(t) \times \Pi^H_{\boldsymbol{M}}(t)$ matrix of M's and zeros.
$\boldsymbol{I}$	A representation of the iridophore layer.	A $\Pi^L_{\boldsymbol{I}}(t) \times \Pi^H_{\boldsymbol{I}}(t)$ matrix of I^l's, I^d's and zeros.
$\Pi^L_D(t)$	Length of domain type $D \in \{\boldsymbol{X}, \boldsymbol{I}, \boldsymbol{M}\}$ in terms of the number of lattice sites at time t.	
$\Pi^H_D(t)$	Height of domain type $D \in \{\boldsymbol{X}, \boldsymbol{I}, \boldsymbol{M}\}$ in terms of the number of lattice sites at time t.	
Δ_D	Site size of domain type $D \in \{\boldsymbol{X}, \boldsymbol{I}, \boldsymbol{M}\}$ in mm.	$\Delta_D = \begin{cases} 0.04 \text{ for } D = \boldsymbol{M}, \\ 0.02 \text{ for } D = \boldsymbol{X}, \boldsymbol{I}. \end{cases}$
$D(i,j,t)$ where $D \in \{\boldsymbol{X}, \boldsymbol{M}, \boldsymbol{I}\}$	Occupancy of row i, column j, of matrix representing domain type D at time t.	$D(i,j,t) = \begin{cases} 0 \text{ or } M, & \text{if } D = \boldsymbol{M} \\ 0, X^b \text{ or } X, & \text{if } D = \boldsymbol{X} \\ 0, I^l \text{ or } I^d, & \text{if } D = \boldsymbol{I} \end{cases}$
t	Simulated time in minutes.	
$\Omega_H(t)$	Simulated height at time t in mm.	$\Omega_H(t) = \Pi^H_D(t) \times \Delta_D \quad \forall D \in \{\boldsymbol{X}, \boldsymbol{I}, \boldsymbol{M}\}$.
$\Omega_L(t)$	Simulated length at time t in mm.	$\Omega_L(t) = \Pi^L_D(t) \times \Delta_D \quad \forall D \in \{\boldsymbol{X}, \boldsymbol{I}, \boldsymbol{M}\}$.
$\Omega_{SL}(t)$	Simulated Standard Length (SL) at time t in mm.	$\Omega_{SL}(t) = \Omega_L(t) + 5.7$.
