# Supplementary material for: A quantitative modelling approach to zebrafish pigment pattern formation
Source: eLife. 2020 Jul 27;9:e52998. doi: 10.7554/eLife.52998 (PMC7384860; doi:10.7554/eLife.52998)
Supplement: Supplementary file 2. [file elife-52998-supp2.pdf]

Supplementary File 2: This table summarises all of the notation used in Appendix 1 regarding short and long range interactions.

Symbol	Description	Definition
$S_D(r,k)$	Set of site positions that are within distance 0.04 mm from site $D(r,k)$ on domain D (within the Moore neighbourhood). See Figures S??(A) and (B) for examples where $D=\boldsymbol{M}$, $D=\boldsymbol{X}$ respectively.	$S_D(r,k)=\begin{cases}\{(i,j)\in(\Pi_D^L,\Pi_D^H)\backslash(r,k)\mid \max\{\min\{\lvert r-i\rvert,\Pi_D^H-\lvert r-i\rvert\},\lvert k-j\rvert\}=1\}, \text{ for } D=\boldsymbol{M}\\ \{(i,j)\in(\Pi_D^L,\Pi_D^H)\backslash(r,k)\mid \max\{\min\{\lvert r-i\rvert,\Pi_D^H-\lvert r-i\rvert\},\lvert k-j\rvert\}\leq 2\} \text{ for } D\neq\boldsymbol{M}\end{cases}$
$L_D(r,k)$	Set of site positions that are distance 0.24mm from site $D(r,k)$ on domain D. See Figures S??(E) and (F) for examples where $D=\boldsymbol{M}$, $D=\boldsymbol{X}$ respectively.	$L_D(r,k)=\begin{cases}\{(i,j)\in(\Pi_D^L,\Pi_D^H)\backslash(r,k)\mid \max\{\min\{\lvert r-i\rvert,\Pi_D^H-\lvert r-i\rvert\},\lvert k-j\rvert\}=6\}, \text{ for } D=\boldsymbol{M}\\ \{(i,j)\in(\Pi_D^L,\Pi_D^H)\backslash(r,k)\mid \max\{\min\{\lvert r-i\rvert,\Pi_D^H-\lvert r-i\rvert\},\lvert k-j\rvert\}=12\} \text{ for } D\neq\boldsymbol{M}\end{cases}$
$S_D^C(r,k,t)$ (Defined only when C lies on D)	Set of site positions distance 0.04mm from site $D(r,k)$ on domain D occupied by cell type C at time t. See Figures S??(C) and (D) for examples where $C=M$, $D=\boldsymbol{M}$, and $C=X$, $D=\boldsymbol{X}$ respectively.	$S_D^C(r,k)=\{(i,j)\in S_D(r,k)\mid D(i,j)=C\}$
$L_D^C(r,k,t)$ (Defined only when C lies on D)	Set of site positions distance 0.24mm from site $D(r,k)$ occupied by cell type C, at time t. See Figures S??(G) and (H) for examples where $C=M$, $D=\boldsymbol{M}$, and $C=X$, $D=\boldsymbol{X}$ respectively.	$L_D^C(r,k)=\{(i,j)\in L_D(r,k)\mid D(i,j)=C\}$
$N_{C_1,C_2}^S(r,k,t)$, where C_1 lies on D_1, C_2 lies on D_2.	Number of cells of type C_2 that are in the short range distance (0.04mm) from site $D_1(r,k)$ where $D_1(r,k)=C_1$ at time t. See Figures S??(A)-(D) for examples where $(C_1,C_2)=(M,M),(M,X),(X,X),(X,M)$ respectively.	$N_{C_1,C_2}^S(r,k,t)=\begin{cases}S_{D_2}^{C_2}(r,k,t) \text{ if } D_1=D_2 \text{ or } D_1 \text{ and } D_2\in\{\boldsymbol{X},\boldsymbol{I}\}.\\ \sum_{(i,j)\in S_{D_1}(r,k)} \mathbb{1}_{\boldsymbol{M}\left(\lceil\frac{i}{2}\rceil,\lceil\frac{j}{2}\rceil\right)=M} \text{ if } D_1\neq\boldsymbol{M},\ D_2=\boldsymbol{M}.\\ \sum_{(i,j)\in S_{\boldsymbol{M}}(r,k)} \mathbb{1}_{D_2(2i,2j)=C_2}+\mathbb{1}_{D_2(2i-1,2j)=C_2}+\mathbb{1}_{D_2(2i,2j-1)=C_2}+\\ \mathbb{1}_{D_2(2i-1,2j-1)=C_2} \text{ if } D_1=\boldsymbol{M},\ D_2\neq\boldsymbol{M}.\end{cases}$
$N_{C_1,C_2}^L(r,k,t)$, where C_1 lies on D_1, C_2 lies on D_2.	Number of cells of type C_2 in long range distance 0.24mm from site $D_1(r,k)$ where $D_1(r,k)=C_2$ at time t. See Figures S??(E)-(H) for examples where $(C_1,C_2)=(M,M),(M,X),(X,X),(X,M)$ respectively.	$N_{C_1,C_2}^L(r,k,t)=\begin{cases}L_{D_2}^{C_2}(r,k,t) \text{ where } D_1=D_2 \text{ or } D_1 \text{ and } D_2\in\{\boldsymbol{X},\boldsymbol{I}\}.\\ \sum_{(i,j)\in L_{D_1}(r,k)} \mathbb{1}_{\boldsymbol{M}\left(\lceil\frac{i}{2}\rceil,\lceil\frac{j}{2}\rceil\right)=M} \text{ where } D_1\neq\boldsymbol{M},\ D_2=\boldsymbol{M}.\\ \sum_{(i,j)\in L_{\boldsymbol{M}}(r,k)} \mathbb{1}_{D_2(2i,2j)=C_2}+\mathbb{1}_{D_2(2i-1,2j)=C_2}+\mathbb{1}_{D_2(2i,2j-1)=C_2}+\\ \mathbb{1}_{D_2(2i-1,2j-1)=C_2} \text{ where } D_1=\boldsymbol{M},\ D_2\neq\boldsymbol{M}.\end{cases}$
w	Weighting factor for comparing the number of M with a different cell type $C\in\{X,I^l,I^d,X^b\}$ in the short range (long range) distance from a site on domain $\boldsymbol{M}(r,k)$. The number of M in this case is multiplied by w to account for the relative size of M to C. See Figures S??(A)-(B), (E)-(F) for examples where this is applicable in the short range, long range respectively.	$w=4$.
$N_C^T(t)$	Total number of cells of type C at time t where cell type C lies on domain D.	$N_C^T(t)=\sum_{i=1}^{\Pi_D^L(t)}\sum_{j=1}^{\Pi_D^H(t)}\mathbb{1}_{D(i,j)=C}$
