# Supplementary material for: A quantitative modelling approach to zebrafish pigment pattern formation
Source: eLife. 2020 Jul 27;9:e52998. doi: 10.7554/eLife.52998 (PMC7384860; doi:10.7554/eLife.52998)
Supplement: Supplementary file 3. — Corresponding dpf for the stages are approximated from the images of Frohnhöfer et al., 2013. [file elife-52998-supp3.pdf]

Supplementary File 3: SL measurements and HAA measurements and WT pattern description by stage are given as by [1]. Corresponding dpf for the stages are approximated from the images of Frohnhöfer *et al* [2].

**Stage**	**SL (mm)**	**HAA (mm)**	**WT pattern description**	**≈ dpf**
PB	7.2	0.83	Faint strip of dense S-iridophores at center	21
PR	8.6	1.2	Loose S-iridophores spreading across 1D and 1V	30
SP	9.6	1.46	Dense S-iridophores present in X1V and X1D	39
SA	10.4	1.7	X1D and X1V forming	44
J	11	2.05	2V and 2D faint	51
J+	13.5	2.5	2V and 2D forming	70
J++	16	3	2V and 2D formed	-

References

[1] D. M. Parichy, M. R. Elizondo, M. G. Mills, T. N. Gordon, and E. Engeszer. Normal Table of Post-Embryonic Zebrafish Development: Staging by Externally Visible Anatomy of the Living Fish. *Dev. Dyn.*, 238(12):2975–3015, 2011.

[2] H. G. Frohnhofer, J. Krauss, H.-M. Maischein, and C. Nusslein-Volhard. Iridophores and their interactions with other chromatophores are required for stripe formation in zebrafish. *Development*, 140(14):2997–3007, 2013.
