# Supplementary material for: A quantitative modelling approach to zebrafish pigment pattern formation
Source: eLife. 2020 Jul 27;9:e52998. doi: 10.7554/eLife.52998 (PMC7384860; doi:10.7554/eLife.52998)
Supplement: Supplementary file 4. [file elife-52998-supp4.pdf]

Supplementary File 4: All continuous time events and their corresponding propensities for event attempts at time $t$, denoted $\alpha_i(t)$ for event $i$ in minutes.

| $i$ | **Event** | **Event description** | $\boldsymbol{\alpha_i(t)}$ | **Assumption based on an average WT fish** |
|---|---|---|---|---|
| 1 | Movement of $M$. | A cell of type $M$ is chosen to attempt movement. | $\frac{0.11N^T_M(t)}{0.04\times60\times24\times7}$ | $M$ move at a rate of 0.11mm per week [1] . |
| 2 | Movement of $X^b$. | A cell of type $X^b$ is chosen to attempt movement. | $\frac{0.033N^T_{X^b}(t)}{0.02\times60\times24\times7}$ | $X^b$ move at a rate of 0.033mm per week [2]. |
| 3 | Movement of $X$. | A cell of type $X$ is chosen to attempt movement. | $\frac{0.033N^T_X(t)}{0.02\times60\times24\times7}$ | $X$ move at a rate of 0.033mm per week [2]. |
| 4 | Movement of $I^d$. | A cell of type $I^d$ is chosen to attempt movement. | $\frac{0.03N^T_{I^d}(t)}{0.02\times60\times24}$ | $I^d$ migrate extensively [3]. We propose that they move at a rate of 0.03mm per day. |
| 5 | Movement of $I^l$. | A cell of type $I^l$ is chosen to attempt movement. | $\frac{0.03N^T_{I^l}(t)}{0.02\times60\times24}$ | $I^l$ migrate extensively [3]. We propose that they move at a rate of 0.03mm per day. |
| 6 | Proliferation of $X$ or $X^b$. | A cell of type $X$ or $X^b$ is chosen to attempt proliferation. | $\frac{N^T_X(t)+N^T_{X^b}(t)}{60\times24\times7}$ | Cells of the xanthophore lineage proliferate continuously from the onset of metamorphosis with a doubling rate of once per week [4]. |
| 7 | Proliferation of $I^d$ or $I^l$. | A cell of type $I^d$ or $I^l$ is chosen to attempt proliferation. | $\frac{1.2N^T_{I^d}(t)}{60\times24} + \frac{1.2N^T_{I^l}(t)}{60\times24}$ | S-iridophores have a doubling time of 3-4 days [5]. We implement that they attempt proliferation 1.2 times per day. |
| 8 | Differentiation of $M$. | A site in $\boldsymbol{M}$ is chosen where a melanocyte progenitor attempts to differentiate into a melanocyte. | $\frac{N^T_{I^d}(t)}{2\times60\times24} + \frac{N^T_X(t)}{10\times60\times24} + \frac{300}{60\times24}$ | Xanthophores and iridophores promote the differentiation of melanocytes in the long range in different strengths [6, 7]. melanocytes also differentiate independently from other cell types [7]. |
| 9 | Differentiation of $X^b$ to $X$. | A cell of type $X^b$ is chosen to attempt differentiation into a cell of type $X$. | $\frac{N^T_{X^b}(t)}{3\times60\times24}$ | We propose that $X^b$ attempt differentiation once every three days. |
| 10 | Transition of $I^d$ to $I^l$. | A cell of type $I^d$ is chosen to attempt transition to a cell of type $I^l$. | $\frac{N^T_{I^d}(t)}{60\times24}$ | We propose that $I^d$ attempt transition once per day. |
| 11 | Transition of $I^l$ to $I^d$. | A cell of type $I^l$ is chosen to attempt transition to a cell of type $I^d$. | $\frac{N^T_{I^l}(t)}{60\times24}$ | We propose that $I^l$ attempt transition once per day. |
| 12 | Melanocyte death. | A cell of type $M$ is chosen to attempt death. | $\frac{N^T_M(t)}{60\times24}$ | We propose that melanocytes may die at least once per day. |
| 13 | Xanthoblast pulls a melanocyte | A cell of type $X^b$ near to cell of type $M$ successfully pulls a cell of type $M$ towards itself. | $\frac{N^T_{X^b}(t)}{100\times60\times24}$ | We propose that this is successful 1/100 times per day. |
| 14 | Growth in the horizontal direction. | The domain is chosen to grow in the horizontal direction. | $\frac{0.13}{0.04\times60\times24}$ | Growth in the horizontal direction is 0.13mm per day [8]. |
| 15 | Growth in the vertical direction. | The domain is chosen to grow in the vertical direction. | $\frac{0.033}{0.04\times60\times24}$ | Growth in the vertical direction is 0.033mm per day [8]. |

# References


[1] G. Takahashi and S. Kondo. Melanophores in the stripes of adult zebrafish do not have the nature to gather, but disperse when they have the space to move. *Pigment Cell Melanoma Res.*, 21(6):677–686, 2008.

[2] H. Yamanaka and S. Kondo. In vitro analysis suggests that difference in cell movement during direct interaction can generate various pigment patterns in vivo. *PNAS*, 111(5):1867–1872, 2014.

[3] A. P. Singh, U. Schach, and C. Nüsslein-Volhard. Proliferation, dispersal and patterned aggregation of iridophores in the skin prefigure striped colouration of zebrafish. *Nat. Cell Biol.*, 16(6):607–14, 2014.

[4] P. Mahalwar, B. Walderich, A. P. Singh, and C. Nüsslein-Volhard. Local reorganization of xanthophores fine-tunes and colors the striped pattern of zebrafish. *Science.*, 345(6202):1362–4, 2014.

[5] B. Walderich, A. P. Singh, P. Mahalwar, and C. Nüsslein-Volhard. Homotypic cell competition regulates proliferation and tiling of zebrafish pigment cells during colour pattern formation. *Nat. Commun.*, 7:11462, 2016.

[6] A. Nakamasu, G. Takahashi, A. Kanbe, and S. Kondo. Interactions between zebrafish pigment cells. *PNAS*, 106(21):8429–8434, 2009.

[7] H. G. Frohnhofer, J. Krauss, H.-M. Maischein, and C. Nusslein-Volhard. Iridophores and their interactions with other chromatophores are required for stripe formation in zebrafish. *Development*, 140(14):2997–3007, 2013.

[8] D. M. Parichy, E. M. Mellgren, J. F. Rawls, S. S. Lopes, R. N. Kelsh, and S. L. Johnson. Mutational analysis of endothelin receptor b1 (rose) during neural crest and pigment pattern development in the zebrafish Danio rerio. *Dev. Bio.*, 227(2):294–306, 2000.
