# Supplementary material for: A quantitative modelling approach to zebrafish pigment pattern formation
Source: eLife. 2020 Jul 27;9:e52998. doi: 10.7554/eLife.52998 (PMC7384860; doi:10.7554/eLife.52998)
Supplement: Supplementary file 5. — Melanocytes, xanthophores, loose iridophores, dense iridophores and xanthoblasts are denoted M, X, Il, Id, Xb respectively. C1, C2 stands for cell 1, cell 2, where C⁢1=X, Xb is the target cell and cell two is the signalling cell with corresponding signal range (R) that is either short (S), up to 0.04 mm, or long (L), 0.12 mm. This signal generates action (A) of cell type C1 of: movement (M), differentiation (D), proliferation (P) or survival (S) of type (T). For action (D) or (S), type (T) is denoted ‘+’ if the resultant action is promotion of action A and ‘-’ if the resultant action is inhibition of action (A). For action (M), type (T) is denoted ‘+’ if the resultant action is attraction towards cell type C1 and ‘-’ if the resultant action is repulsion away from cell type C1. *Melanocytes can also differentiate randomly, independent of any other cell type. [file elife-52998-supp5.pdf]

Supplementary File 5: Full description of all the cell-cell interaction given in main text. All interactions are referenced in Appendix 1. Melanocytes, xanthophores, loose iridophores, dense iridophores and xanthoblasts are denoted M, X, I^l, I^d, X^b respectively. C1, C2 stands for cell 1, cell 2, where $C1 = X$, X^b is the target cell and cell 2 is the signalling cell with corresponding signal range (R) that is either short (S), up to 0.04mm, or long (L), 0.12mm. This signal generates action (A) of cell type C1 of: movement (M), differentiation (D), proliferation (P) or survival (S) of type (T). For action (D) or (S), type (T) is denoted '+' if the resultant action is promotion of action A and '-' if the resultant action is inhibition of action (A). For action (M), type (T) is denoted '+' if the resultant action is attraction towards cell type C1 and '-' if the resultant action is repulsion away from cell type C1. *Melanocytes can also differentiate randomly, independent of any other cell type.

**C1**	**C2**	**A**	**R**	**T**	**Basis of assumption**	**Details**	**Ref.**
I^l	I^l	M	S	-	Adult *pfe*, *shd*, *nac* and WT fish.	I^l are typically spaced at a distance from each other.	[1]
X	I^d	M	S	+	WT fish development.	Arrival of I^d leads to compaction of X overlaying this region.	[2]
I^d	X	M	S	+	Adult *pfe*, *shd*, *nac* and WT fish.	X and I^d are highly associated with each other.	[1]
M	I^d	M	S	-	Long term imaging of WT fish	M appear to migrate to dense I^d free zones.	[3]
I^d	M	M	S	-	Adult *pfe*, *shd*, *nac* and WT fish.	I^d are typically not associated with M.	[1]
X	M	M	S	+	Cultured WT M and X.	*In vitro*, X extended pseudopodia towards M which they then followed towards M.	[4]
M	X	M	S	-	Regeneration experiments performed *in vivo* on WT fish	When X in close proximity to M in the pale interstripes, M move out of interstripe, versus when the X in close proximity to M are ablated. Then M are less likely to leave the interstripe region.	[5]
M	X^b	M	L	+	Long term imaging of WT fish	X^b in stripes exhibit airinemes which attach to M activating Delta-Notch signalling and resulting in the consolidation of M into stripes.	[6, 7]
I^d	M	D	S	-	Observations of WT fish.	M typically differentiate in I^d free zones.	[3]
I^d	M	D	L	+	Observations of *pfe* mutants and WT	M differentiation is associated with one stripe width distance away from the central strip of I^d.	[1]
X	M	D	S	-	Regeneration experiments *in vivo* on WT fish.	M and X compete to differentiate in the S range.	[4]
X	M	S	L	+	Regeneration experiments *in vivo* on WT fish.	Increased M death when X ablated from neighbouring pale interstripes.	[4, 8]
X	M	S	S	-	Regeneration experiments *in vivo* on WT fish.	When X in close proximity to M, some M die versus when X in close proximity to M are ablated, no M die.	[5, 9]
X	M	D	L	+	Regeneration experiments *in vivo* on WT fish.	M regeneration proportional to neighbouring pale interstripe X number.	[4, 8]

C1	C2	A	R	T	Basis of assumption	Details	Ref.
M	M	D	L	-	Regeneration experiments *in vivo* on WT fish.	M regeneration negatively proportional to M at half a stripe width distance.	[4]
M	M	S	L	-	Regeneration experiments *in vivo* on WT fish.	M regeneration negatively proportional to M at half a stripe width distance (when X ablated from neighbouring interstripes).	[4]
I^l	M	S	S	+	Observations of *pfe* fish.	We predict that loose S-iridophores promote M survival as this would account for the aggregates of Ms observed in *pfe*.	[10, 1]
*	M	D			Observation of double mutant *nac;pfe* fish.	*nac;pfe* are unable to generate Xs or iridophores, yet readily generate M.	[1]
X^b	I^d	D	S	+	S-iridophore expression studies.	I^d express xanthogenic Colony stimulating factor-1 (Csf1) which is essential for X^b differentiation.	[3, 1, 11]
X^b	X	P	S	+	Regeneration experiments *in vivo* on WT fish.	M and X compete to differentiate in the short range.	[4]
X^b	M	D	S	-	Regeneration experiments *in vivo* on WT fish.	M and X compete to differentiate in the short range.	[4]
X	I^d	P	S	+	S-iridophore expression studies.	I^d express xanthogenic Colony stimulating factor-1 (Csf1) which is essential for X proliferation and survival.	[3, 1, 11]
X	M	P	S	-	Regeneration experiments *in vivo* on WT fish.	M and X compete to differentiate in the short range.	[4]
X	X	P	S	+	Regeneration experiments *in vivo* on WT fish.	M and X compete to differentiate in the short range.	[4]
X		P			Clonal analyses of X lineage.	X and X^b observed to proliferate at a rate of approximately 1/week.	[2]
X^b.		P			Clonal analyses of X lineage.	X and X^b observed to proliferate at a rate of approximately 1/week.	[2]

References

[1] H. G. Frohnhofer, J. Krauss, H.-M. Maischein, and C. Nusslein-Volhard. Iridophores and their interactions with other chromatophores are required for stripe formation in zebrafish. *Development*, 140(14):2997–3007, 2013.

[2] P. Mahalwar, B. Walderich, A. P. Singh, and C. Nüsslein-Volhard. Local reorganization of xanthophores fine-tunes and colors the striped pattern of zebrafish. *Science.*, 345(6202):1362–4, 2014.

[3] L. B. Patterson and D. M. Parichy. Interactions with Iridophores and the Tissue Environment Required for Patterning Melanophores and Xanthophores during Zebrafish Adult Pigment Stripe Formation. *PLoS Genet.*, 9(5), 2013.

[4] A. Nakamasu, G. Takahashi, A. Kanbe, and S. Kondo. Interactions between zebrafish pigment cells. *PNAS*, 106(21):8429–8434, 2009.

[5] G. Takahashi and S. Kondo. Melanophores in the stripes of adult zebrafish do not have the nature to gather, but disperse when they have the space to move. *Pigment Cell Melanoma Res.*, 21(6):677–686, 2008.

[6] D. S. Eom, E. J. Bain, L. B. Patterson, M. E. Grout, and D. M. Parichy. Long-distance communication by specialized cellular projections during pigment pattern development and evolution. *eLife*, 4(December 2015):1–25, 2015.

[7] D. S. Eom and D. M. Parichy. A macrophage relay for long-distance signaling during postembryonic tissue remodeling. *Science*, 355(6331):1317–1320, 2017.

[8] S. Kondo and M. Watanabe. Black, yellow, or silver: Which one leads skin pattern formation? *Pigment Cell Melanoma Res.*, 28(1):2–4, 2015.

[9] H. Yamanaka and S. Kondo. In vitro analysis suggests that difference in cell movement during direct interaction can generate various pigment patterns in vivo. *PNAS*, 111(5):1867–1872, 2014.

[10] C. B. Kimmel, W.W. Ballard, S. R. Kimmel, B. Ullmann, and T. F. Schilling. Stages of embryonic development of the zebrafish. *Dev. Dyn.*, 203(3):253–310, 1995.

[11] B. Walderich, A. P. Singh, P. Mahalwar, and C. Nüsslein-Volhard. Homotypic cell competition regulates proliferation and tiling of zebrafish pigment cells during colour pattern formation. *Nat. Commun.*, 7:11462, 2016.
