# Supplementary material for: A quantitative modelling approach to zebrafish pigment pattern formation
Source: eLife. 2020 Jul 27;9:e52998. doi: 10.7554/eLife.52998 (PMC7384860; doi:10.7554/eLife.52998)
Supplement: Supplementary file 6. [file elife-52998-supp6.pdf]

Supplementary File 6: Parameters implemented in the model.

**Sym.**	**Value**	**Units**	**Description**	**Reference**
L	0.24	mm	Long range cell-cell (filopodia) interaction radius.	Long interactions are approximately the length of half of the adult stripe width [1].
S	0.4	mm	Short range cell-cell interaction radius.	Short range interactions are with neighbouring cells.
a_{MM}	0	None	Attraction bias M to M.	Melanocytes neither attract nor repulse other melanophores [2].
a_{MX}	0	None	Attraction bias M to X.	Melanocytes are repelled by xanthophores in the short range [2, 3].
a_{MI^d}	0	None	Attraction bias M to I^d.	Melanocytes and iridophores mutually repulse each other in the short range [4].
r_{MM}	0	None	Repulsion bias M to M.	Melanocytes neither attract nor repel other melanocytes [2].
r_{MX}	100	None	Repulsion bias M to X.	Melanocytes are repelled by xanthophores in the short range [2, 3].
r_{MI^d}	30	None	Repulsion bias M to I^d.	Melanocytes and iridophores mutually repel each other in the short range [4].
a_{XM}	10	None	Attraction bias X to M.	Xanthophores 'chase' melanocytes in the short range [3].
a_{XX}	0	None	Attraction bias X to X.	No known interaction between xanthophores.
a_{XI^d}	1000	None	Attraction bias X to I^d.	Iridophores attract xanthophores in the short range [4].
r_{XM}	5	None	Repulsion bias X to M.	Xanthophores 'chase' melanocytes in the short range [3].
a_{I^dX}	300	None	Attraction bias I^d to X.	Predict that xanthophores mutually promote the consolidation of dense iridophores in the interstripe.
$a_{I^dI^d}$	0	None	Attraction bias I^d to I^d.	No known interaction between dense iridophores.
r_{I^dM}	10	None	Repulsion bias I^d to M.	Melanocytes and iridophores mutually repel each other in the short range [4].
r_{I^dX}	0	None	Repulsion bias I^d to X.	Predict that xanthophores mutually promote the consolidation of dense iridophores in the interstripe.
$r_{I^dI^d}$	0	None	Repulsion bias I^d to I^d.	No known interaction between dense iridophores.
α	2.5	None	Scaling factor for melanocyte differentiation criteria.	Chosen so that melanocyte differentiation is only successful when there are more X and I^d in the long range than M.
β	3	None	Lower bound for melanocyte differentiation (minimum number of X and I^d in the long range.	Chosen so that melanocyte differentiation is only successful when there are some X and I^d in the long range.
γ	3	None	Scaling factor for melanocyte differentation (numbers of X vs number of M in short range).	Chosen so that melanocyte differentiation is only successful when X does not outcompete M in the short range.
κ	3	None	Upper bound of I^d in the short range for melanocyte differentation.	Chosen so that melanocyte differentiation is only successful when I^d is limited in the short range.
L_x	12	None	Lower bound for xanthophore number in the long range to promote iridophore transition from dense to loose.	Optimised for stripe formation.
S_x	9	None	Upper bound for xanthophore number in the short range to promote iridophore transition from dense to loose.	Optimised for stripe formation.
S_m	1	None	Lower bound for melanocyte number in the short range to promote iridophore transition from dense to loose.	Optimised for stripe formation.
L_{x2}	16	None	Upper bound for xanthophore number in the long range to promote iridophore transition from loose to dense.	Optimised for stripe formation.
S_{x2}	4	None	Lower bound for xanthophore number in the short range to promote iridophore transition from loose to dense.	Optimised for stripe formation.
S_{m2}	12	None	Lower bound for melanocyte number in the short range to promote iridophore transition from loose to dense.	Optimised for stripe formation.
σ	3	None	Scaling factor for melanocyte death (number of M compared to number of X.	Chosen so that M die when they are surrounded by X.
ω	3		Lower bound for number of I^l in the short range to promote M death.	Chosen so that M die when they are surrounded by I^l.
h	5	cell diameters	Aireneme length (used in determining set P).	Airenemes reach up to several cell diameters (>0.15 mm). [5]

References

[1] A. Nakamasu, G. Takahashi, A. Kanbe, and S. Kondo. Interactions between zebrafish pigment cells. *PNAS*, 106(21):8429–8434, 2009.

[2] G. Takahashi and S. Kondo. Melanophores in the stripes of adult zebrafish do not have the nature to gather, but disperse when they have the space to move. *Pigment Cell Melanoma Res.*, 21(6):677–686, 2008.

[3] H. Yamanaka and S. Kondo. In vitro analysis suggests that difference in cell movement during direct interaction can generate various pigment patterns in vivo. *PNAS*, 111(5):1867–1872, 2014.

[4] L. B. Patterson and D. M. Parichy. Interactions with Iridophores and the Tissue Environment Required for Patterning Melanophores and Xanthophores during Zebrafish Adult Pigment Stripe Formation. *PLoS Genet.*, 9(5), 2013.

[5] D. S. Eom and D. M. Parichy. A macrophage relay for long-distance signaling during postembryonic tissue remodeling. *Science*, 355(6331):1317–1320, 2017.
